# Chlorophyll-binding proteins revisited - a multigenic family of light-harvesting and stress proteins from a brown algal perspective

**DOI:** 10.1186/1471-2148-10-365

**Published:** 2010-11-26

**Authors:** Simon M Dittami, Gurvan Michel, Jonas Collén, Catherine Boyen, Thierry Tonon

**Affiliations:** 1 UPMC Univ Paris 06, UMR 7139 Marine Plants and Biomolecules, Station Biologique, 29680, Roscoff, France; 2 CNRS, UMR 7139 Marine Plants and Biomolecules, Station Biologique, 29680, Roscoff, France

## Abstract

**Background:**

Chlorophyll-binding proteins (CBPs) constitute a large family of proteins with diverse functions in both light-harvesting and photoprotection. The evolution of CBPs has been debated, especially with respect to the origin of the LI818 subfamily, members of which function in non-photochemical quenching and have been found in chlorophyll a/c-containing algae and several organisms of the green lineage, but not in red algae so far. The recent publication of the *Ectocarpus siliculosus *genome represents an opportunity to expand on previous work carried out on the origin and function of CBPs.

**Results:**

The *Ectocarpus *genome codes for 53 CBPs falling into all major families except the exclusively green family of chlorophyll a/b binding proteins. Most stress-induced CBPs belong to the LI818 family. However, we highlight a few stress-induced CBPs from *Phaeodactylum tricornutum *and *Chondrus crispus *that belong to different sub-families and are promising targets for future functional studies. Three-dimensional modeling of two LI818 proteins revealed features common to all LI818 proteins that are likely to interfere with their capacity to bind chlorophyll b and lutein, but may enable binding of chlorophyll c and fucoxanthin. In the light of this finding, we examined the possibility that LI818 proteins may have originated in a chlorophyll c/fucoxanthin containing organism and compared this scenario to three alternatives: an independent evolution of LI818 proteins in different lineages, an ancient origin together with the first CBPs, before the separation of the red and the green lineage, or an origin in the green lineage and a transfer to an ancestor of haptophytes and heterokonts during a cryptic endosymbiosis event.

**Conclusions:**

Our findings reinforce the idea that the LI818 family of CBPs has a role in stress response. In addition, statistical analyses of phylogenetic trees show an independent origin in different eukaryotic lineages or a green algal origin of LI818 proteins to be highly unlikely. Instead, our data favor an origin in an ancestral chlorophyll a/c-containing organism and a subsequent lateral transfer to some green algae, although an origin of LI818 proteins in a common ancestor of red and green algae cannot be ruled out.

## Background

Photosynthesis is a central process in plant physiology, which involves the collection of solar energy *via *two types of light-harvesting complexes (LHC-I and LHC-II). LHC-II is the most abundant of these complexes in thylakoid membranes, and consists of pigments (chlorophylls and carotenoids) which are bound to chlorophyll-binding proteins (CBPs). LHC proteins constitute a large family of proteins [1,2], which includes chlorophyll a/b-binding proteins (CABs), fucoxanthin chlorophyll a/c-binding proteins (FCPs) [3,4], high light-induced proteins (HLIPs), early light-induced proteins (ELIPs), the psbS subunit of photosystem II (psbS), and stress-enhanced proteins (SEPs). CABs, as well as FCPs (together referred to as CBPs in this manuscript), have been suggested to have emerged from cyanobacterial HLIPs as a result of two duplication events and the subsequent loss of one transmembrane helix [1], but this evolutionary scenario has been recently challenged by Engelken et al. [5]. CABs and FCPs were frequently reported to be transcriptionally repressed in response to light stress [6-8].

Although CBPs are classically considered as light-harvesting proteins, increasing amounts of data point to possible additional functions within this protein family. For example, a number of genes encoding FCPs were observed to be down-regulated in a developmental mutant of the brown alga *Ectocarpus siliculosus *[9]. Moreover, several recent transcriptomic studies of stress response [10-14] highlighted FCPs that were up-regulated in response to heat-, salt-, oxidative-, or light stress in both brown algae and diatoms. Within the green lineage, similar observations were made concerning CBPs in *Chlamydomonas reinhardtii *after high light treatment [15,16]. The up-regulated CBPs were referred to as stress-induced CBPs, LI818 proteins, or LHCSR [15,17,18]. They were shown to constitute one of several distinct families of LHC proteins [14,19-21], and can be found in a range of eukaryotic lineages.

A role of these proteins in light-harvesting seems improbable considering their expression profiles. Functional analyses of both stress-induced CBPs and closely related non stress-induced CBPs carried out in a few model eukaryotes suggest that at least some of these proteins function in non-photochemical quenching (NPQ), *i.e. *the dissipation of excess light energy. In *C. reinhardtii*, the LI818 polypeptide, unlike other CBPs, was shown to be only loosely embedded in the thylakoid membranes and to be localized in stroma-exposed regions [22], and mutants of this protein were affected in their capacity to adapt to high light [18]. Paralogs of LI818 proteins in the moss *Physcomitrella patens *(named LHCSRs), even though not stress-induced on a transcriptomic level, were also shown to be active in promoting NPQ and to contribute to photoprotection under high light conditions [23]. Moreover, in the diatom *Cyclotella meneghiniana*, proteins of this family have been suggested to bind diadinoxanthin and diatoxanthin [24], and are important to quench fluorescence [25]. In another diatom, *P. tricornutum*, a LI818 protein has been shown to be required for efficient light response and to influence natural variability in photoresponse [26]. Similar suggestions have also been made for LI818 proteins from the haptophyte *Emiliana huxlei *[21].

In addition to uncertainties about the function of these LI818 and LI818-like proteins, their evolutionary origin has not yet been finally resolved. Neilson and Durnford [20] argued that, since LI818 and LI818-like proteins have been identified in diverse groups of photosynthetic organisms, they are likely to have been amongst the first eukaryotic light-harvesting proteins. On the other hand, several reports have suggested that these proteins have evolved within the green lineage and were transferred to the heterokonts and other chlorophyll a/c-containing algae during a cryptic primary endosymbiosis event, which involved the uptake of a green algal endosymbiont by an ancestral chromalveolate [18,27,28]. However, this latter theory, as well as the chromalveolate hypothesis in general, is highly debated [29-32], particularly because the only genomic data on red algae come from an atypical alga with a reduced genome and without cell wall [33].

The recent publication of the genome of the brown alga *Ectocarpus siliculosus *[34] represents an interesting opportunity to expand on previous work carried out on the origin and function of CBPs. The *Ectocarpus *genome codes for a total of 53 CBPs belonging to different families [34], several of which have already been shown to be induced in response to stress [12]. Taking into consideration the new sequence data from *E. siliculosus*, we aimed to explore both the diversity and evolution of this large family of proteins, focusing our attention on stress-induced CBPs. First, we examined the phylogenetic position of known stress-induced CBPs from different organisms. We then sought to assess structural and evolutionary differences between CABs and the LI818 family, the latter comprising most of the stress-induced CBPs. To this means, homology modeling and structural superimposition of *E. siliculosus *and *C. reinhardtii *proteins belonging to the LI818 clade with a previously crystallized spinach CAB were performed. Finally, several hypotheses on the origin of the LI818 family were tested.

## Results and discussion

### Genomes of photosynthetic haptophytes and heterokonts code for high numbers of CBPs and LI818 family proteins

**Figure 1:**
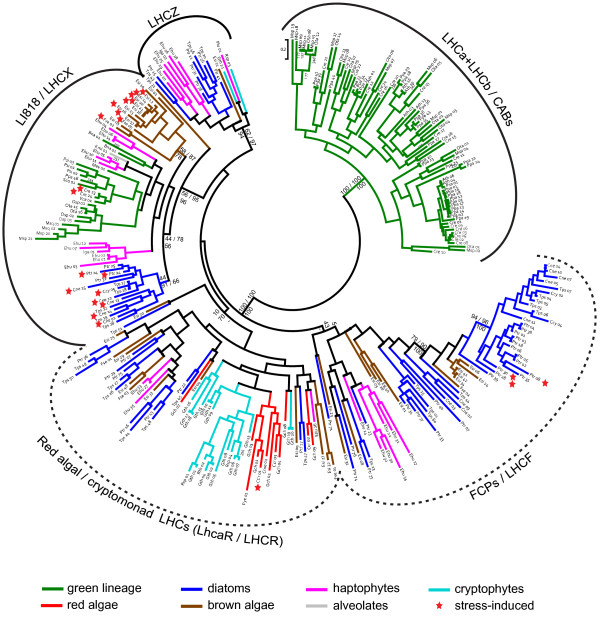
**Phylogenetic tree of CBPs**. Red stars next to the sequence name indicate genes that have been shown to be induced in response to stress. Only selected confidence values were plotted (PhyML bootstrap, PhyML Approximate Likelihood test, and MrBayes posterior probabilities respectively, with dash indicating no support by MrBayes). Dotted lines were used to indicate groups according to current naming conventions, but which are poorly resolved in our phylogeny (see text)*. Abbreviations: *Ath *= Arabidopsis thaliana*, Bna *= Bigelowiella natans*, Ccr *= Chondrus crispus*, Cme *= Cyanidioschyzon merolae*, Cne *= Chaetoceros neogracile*, Cre *= Chlamydomonas reinhardtii*, Ccy *= Cyclotella meneghiniana*, Ehu *= Emiliania huxleyi*, Esi *= Ectocarpus siliculosus*, Fse *= Fucus serratus*, Fve *= Fucus vesiculosus*, Gch *= Gracilaria changii*, Gth = *Guillardia theta*, Iga *= Isochrysis galbana*, Kmi *= Karlodinium micrum*, Mev *= Mesostigma viride*, Msp *= Micromonas sp. RCC299*, Msq = *Micromonas sp. *CCMP490, Osp = *Ostreococcus sp*. RCC809, Ota *= Ostreococcus tauri*, Plu = *Pavlova lutheri*, Ppa *= Physcomitrella patens*, Psa *= Pisum sativum*, Psi = *Picea sitchensis*, Ptr *= Phaeodactylum tricornutum*, Pye *= Porphyra yezoensis*, Rsp = *Rhodomonas sp. CS24*, Sco *= Scenedesmus obliquus*, Tps *= Thalassiosira pseudonana*, Vca = *Volvox carteri.*

An extensive search for CBPs in fully sequenced genomes revealed that, on average, heterokonts and haptophytes contain the highest total numbers of CBPs (Table 1, Additional file 1), although, within the green lineage, *Volvox cateri *and *Physcomitrella patens *also possess numerous copies of these proteins. LI818 family proteins are only scarcely represented within the green lineage. They were found in one to three copies in several green algae, in the genome of the moss *P. patens*, and in EST libraries produced for gymnosperms, notably *Picea glauca *(two copies, gb|DR591434.1 and gb|CO250289.1) and *Picea sitchensis *(three copies, Figure 1). Incomplete copies were also found in the angiosperms *Medicago truncatula *(one copy, gb|BG452558.1) and *Quercus robur *(one copy, gb|FR633552.1). In the genome of the red alga *Cyanidioschyzon merolae *and EST libraries for *Chondrus crispus *(4,114 ESTs)*, Porphyra yezoensis *(20,069 ESTs), and *Gracilaria changii *(8,147 ESTs), no gene encoding any LI818 protein was identified; neither was any homolog found among the available sequences for the cryptophytes *Guillardia theta *(15,173 ESTs) and *Rhodomonas sp. *[35]. In contrast, numerous proteins of this family were present in haptophytes and heterokonts, where 4 to 15 copies were found in the examined genomes. These data indicate the importance of CBPs, and in particular of LI818 proteins, in the marine environment and among haptophytes and heterokonts.

**Table 1:** Total number of proteins containing a chlorophyll a/b-binding domain (PFAM00504/IPR022796) in a selection of eukaryotic genomes, as well as the number of CBPs belonging to the LI818 family.

Organism	Total number	LI818 family proteins	Genome size (Mbp)
*Arabidopsis thaliana*	22	0	125
*Populus trichocarpa*	23	0	480
*Oryza sativa*	16	0	206
*Selaginella moellendorffii*	12	0	213
*Physcomitrella patens*	47	2	480
*Ostreococcus lucimarinus*	16	1	13
*Ostreococcus tauri*	15	1	13
C*hlamydomonas reinhardtii*	25	3	112
*Micromonas sp*. RCC299	21	2	21
*Coccomyxa sp. *C-169	24	1	49
*Chlorella sp. *NC64A	20	1	46
*Volvox cateri*	37	1	140
*Phaeodactylum tricornutum*	40	4	26
*Thalassiosira pseudonana*	40	5	32
*Ectocarpus siliculosus*	53	13	200
*Emiliania huxleyi*	87	15	168
*Cyanidioschyzon merolae*	3	0	16

Accession numbers for each of the proteins are given in Additional file 1.

### CABs and FCPs contain several subfamilies

In order to establish relationships between the *E. siliculosus *sequences and other LHC proteins identified in chlorophyll a/b-, chlorophyll a-, and chlorophyll a/c-containing organisms, phylogenetic analyses with CBPs from a wide range of taxa were performed. The topology of the tree presented in Figure 1 is similar to results previously published [14,19-21]. Two groups are clearly distinguishable. A first one comprised sequences only from the green lineage and contained most of the CABs from terrestrial plants (LHCa + LHCb). It will not be further discussed here, because it was already considered in detail in previous articles [19,36]. The remaining part of the tree constitutes the second group, which can be split into several major divisions, each comprising sequences from photosynthetic heterokonts and haptophytes, and containing a certain number of subfamilies and taxon-specific subgroups.

The first division to consider was originally highlighted by Koziol et al. [19] and was composed of members from the cryptomonads, haptophytes, and chlorarachniophytes. It was named LHCZ in absence of any indication on function or localization of this class of proteins. In our phylogeny, LHCZ also contained one brown algal as well as several diatom sequences.

The second division corresponds to the LI818 family (also named LHCX in diatoms, [10,11,13]), and contains most of the stress-induced CBPs. It features three subgroups with moderate statistical support, which correspond to different taxonomic groups. One is formed almost exclusively by stress-induced genes from several species of diatoms, and contains the recently functionally characterized PtLHCX1 [26] (Ptr_25 in our phylogeny). Another one comprises several *E. siliculosus *sequences, many of which were induced in response to abiotic stress conditions and most of them are likely to have been subject to concerted evolution and/or to result from recent duplications. Effectively, proteins Esi_12 to Esi_22 are all highly similar and located in close proximity on supercontig 0085 in linkage group 16 of the *Ectocarpus *genome [37]. This group also contains one *Fucus *sequence. The third subgroup is the most heterogeneous and comprises sequences belonging mainly to haptophytes and to organisms of the green lineage. This subgroup also contains the LI818 proteins that have been recently functionally characterized in *Chlamydomonas *and in *Physcomitrella*, and which function in NPQ.

The remaining sequences were previously divided into two groups [19,20]: one named cryptomonad/red algal LHC clade [14,19], LhcaR [20], and LHCR in diatoms [13,38], and a second one named FCP or LHCF clade [19,20]. However, in this study as well as in the study of Neilson and Durnford [20], statistical support for the separation of these two subgroups is insufficient (10 and 5 for PhyML/bootstrap, 70 and 43 for PhyML/SH test, and no support by MrBayes). Moreover, the position of these two sub-groups with respect to the LI818 and LHCZ divisions is poorly resolved in both studies [20]. We mention these divisions in our study because they correspond to current naming conventions, but the biological relevance of such a distinction is questionable. In contrast, among the LHCF sequences, we identified a well-supported subgroup of proteins containing sequences from several species of diatoms, three of which have been shown to be induced in response to light stress [13], as well as a group of closely related *Ectocarpus *sequences.

### Stress-induced chlorophyll-binding proteins

One of the questions we attempted to answer in this study was whether all stress-induced CBPs, both in *Ectocarpus *and in other photosynthetic organisms, fall into the same clade. Figure 1 clearly demonstrates that most of the stress-induced CBPs from most examined organisms (all for *Ectocarpus*) belong to the LI818 clade. This is in agreement with the supposed function of these proteins in NPQ [18,23,25]. Nevertheless, not all LI818 proteins have been shown to be transcriptionally induced in response to stress. This can be seen for example in the case of the moss *P. patens*, where neither of the two known LI818 proteins (represented by Ppa_46 in Figure 1) were up-regulated in response to osmotic-, salt-, drought, and UV-B stress in two independent studies [39,40]. Another recent study in *P. tricornutum *[26] has shown the protein LHCX1 to be induced at a protein level in response to high light, although no changes were observed on the mRNA level.

In addition to non stress-induced LI818 proteins, we also found two cases of stress-induced CBPs from other clades. The first of these cases are high light-induced CBPs from *P. tricornutum*. While four of them clearly fell into the LI818 clade, forming a subgroup with other diatom sequences, three others fell into the LHCF group and are part of a strongly supported group of diatom proteins with a sister group of *E. siliculosus *proteins. The second case is the red algal protein Ccr_01, which fell into the LhcaR clade and which was strongly induced in response to different abiotic stresses: 87-fold induced in high temperature, 21-fold in high light, and 3.6-fold in low salinity (p < 0.05 for all treatments) [41]. This finding is particularly interesting considering that in public red algal EST databases or in the (strongly reduced) genome of *Cyanidioschyzon*, we were not able to find either LI818 or psbS proteins. Should the absence of these proteins be confirmed by the ongoing *Chondrus *and *Porphyra *genome projects, Ccr_01 could be an excellent candidate for an NPQ-regulating protein in red algae. In any case, the discovery of stress-induced CBPs in different families supports the hypothesis that functions other than light-harvesting may have evolved independently in different CBP clades and in different organisms.

### Modeling of LI818 family proteins from *Chlamydomonas reinhardtii *and *Ectocarpus siliculosus*

After having observed that most of the stress-induced CBPs belong to the LI818 family, we attempted to assess the structural and evolutionary changes that have occurred in LI818 proteins compared to CABs by homology modeling of two sequences of this subfamily, one representative for the "brown" lineage (Esi_02 from *E. siliculosus*) and another for the "green" lineage (Cre_23 from *C. reinhardtii*). The N- and C-terminal ends of Cre_23 and Esi_02 were not included in the models because they are too divergent, and only the residues corresponding to residues Pro 19 to Asp 215 of the spinach CAB were considered. The modeled CBPs from *C. reinhardtii *and *E. siliculosus *display 58% and 60% sequence similarity, respectively, with the spinach CAB, for which the three-dimensional structure has been solved [42]. This similarity level was sufficient to generate a reliable 3D model for Cre_23 and Esi_02.

**Figure 2:**
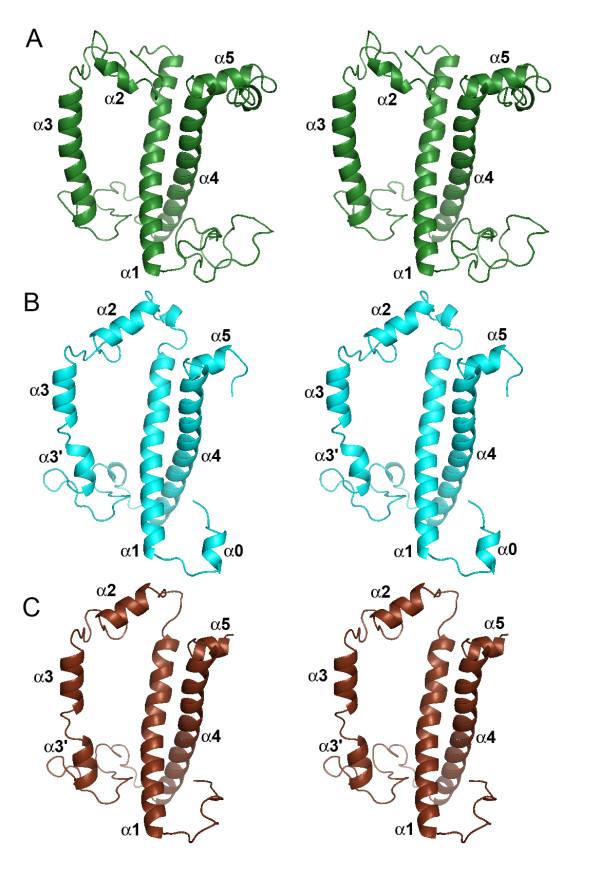
**Structural comparison of the crystallized CAB from spinach with the 3D models of the LI818 protein Cre_23 from *Chlamydomonas reinhardtii *and of the stress-induced LHC protein Esi_02 from *Ectocarpus siliculosus***. Stereo ribbon representation of the crystal structure of the spinach CAB (PDB code: 1RWT) (A), and of the modeled proteins Cre_23 (B) and Esi_02 (C).

**Figure 3:**
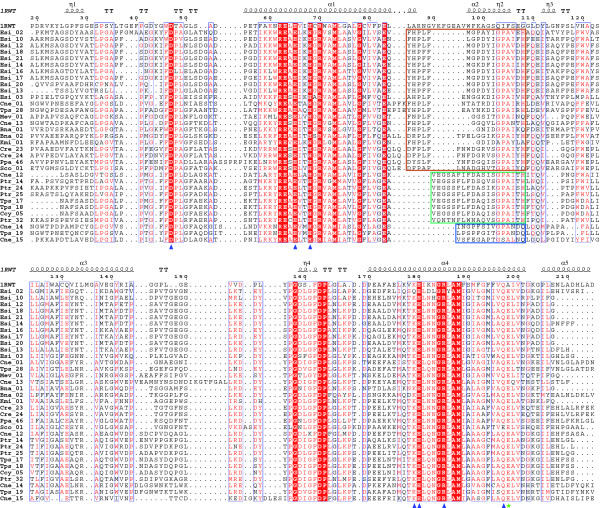
**Structure-based sequence alignment of the crystallized spinach CAB (code **1RWT**) with proteins belonging to the LI818 clade**. The secondary structure of the spinach CAB is shown above the alignment. Conserved amino acids highlighted by a red background are identical and those in red letters are similar. Alpha helices are represented as helices, and *β*-turns are marked with TT. Blue triangles indicate the conserved residues involved in the binding of chlorophyll a molecules. The green star shows the conserved glutamate in LI818-like proteins, predicted to preclude the binding of Chl*b *607 observed in the spinach CAB. The colored frames indicate the three subgroups of helix α2 within the LI818 subfamilies.

The α-helices α1, α3 and α4 and most of the β-turns are well conserved for these proteins (Figure 2), which was confirmed by the multiple sequence alignment of the LI818-like proteins (Figure 3). In these regions, LI818-like proteins feature key residues strictly conserved with the spinach CAB: Asp47 (Asp28, Esi_02 numbering), Glu65 (Glu48), His68 (His51), Glu180 (Glu158), Arg185 (Arg163) and Gln197 (Gln175). The lysine 179 is also well conserved or replaced by polar glutamine. Almost all of these residues are involved in direct binding of chlorophyll a molecules (Chl*a *602, 603, 610, 612, and 613). The arginine 185 stabilizes Glu65 through an ionic interaction, which coordinates the magnesium ion of Chl*a *602 [42].

**Figure 4:**
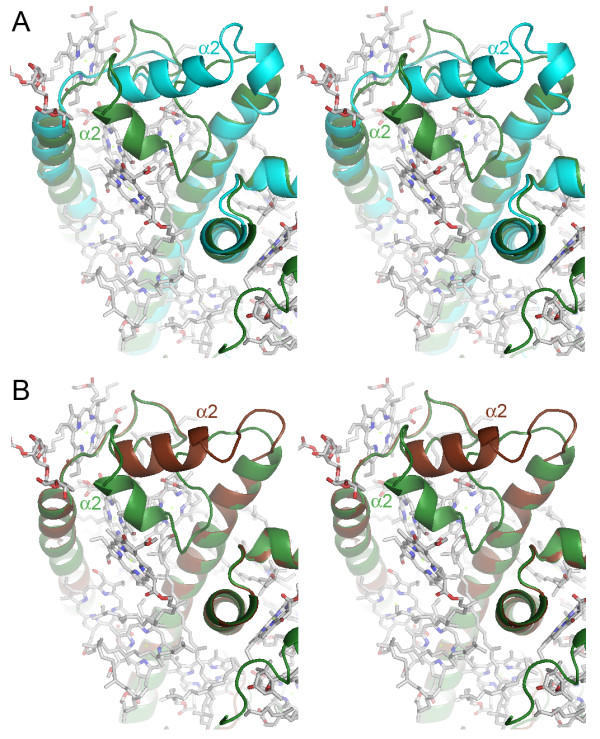
**Comparison of the binding site of chlorophyll b and lutein molecules**. (A) Stereo representation of the superimposition of the crystallized spinach CAB (green) and the modeled LI818 protein Cre_23 from *Chlamydomonas reinhardtii *(cyan). (B) Stereo representation of the superimposition of the crystallized spinach CAB (green) and the modeled LHC Esi_02 (brown). The view is a zoom on the region comprising the helix α2. The chlorophyll and lutein molecules bound to the spinach CBP are represented in balls and sticks, with the CPK color code.

The main difference in the secondary structure is observed in the loop connecting the helices α1 and α2 and in the helix α2 itself. This region is shorter in the LI818 proteins (between 6 and 12 residues shorter) and the sequence composition is completely different (Figure 3), resulting in a significant displacement of the helix α2 in the models of Cre_23 and Esi_02 (Figure 4). All LI818 proteins feature a conserved signature G-P-A-X-X-[H/Q] in this region. The N-terminal end of this loop is more divergent and the sequences can be grouped according to three types of sequence patterns (Figure 3). These three groups roughly correspond to several subfamilies that could be distinguished within the LI818 clades (Figure 1). The group including all LI818 proteins from *E. siliculosus *also comprises some CBPs from green algae (Cre_23, Cre_24, and Sco_01) and from moss (Ppa_46). In the spinach CAB, the helix α2 is involved in the binding of two chlorophyll b molecules (Chl*b *607 and Chl*b *606) and one lutein molecule (Lut 621) [42]. As shown in our 3D models and in the alignment, the location of helix α2 in LI818-like proteins is completely different and cannot provide similar binding interactions for these chlorophyll b and lutein molecules. Moreover, LI818 proteins possess a conserved glutamate residue (Glu176, Esi_02 numbering) instead of an alanine at this position in the spinach CAB (Ala198). Such a substitution would result in a steric clash with the Chl*b *607, suggesting that this molecule is likely absent in LI818 proteins. These structural predictions are consistent with the fact that CBPs from heterokonts and haptophytes do not bind chlorophyll b but bind chlorophyll c. Similarly, brown algal LHCs contain fucoxanthin, instead of lutein. In the spinach CAB, one end of the lutein is bound to Asp47, while the opposite end interacts with Trp97, which is localized in helix α2. In the LI818-like proteins, Asp47 is strictly conserved, but Trp97 is missing, since the equivalent region is completely altered (Figure 3). In addition, fucoxanthin is a shorter carotenoid than lutein, suggesting that one end of a fucoxanthin molecule could interact with Asp47 (Asp28, Esi_02 numbering), but that the recognition of the other end differs from the spinach protein structure. In the Cre_23 model, the helix α2 adopts a conformation similar to the Esi_02 helix α2 (Figure 4), as predicted by the conservation of the signature G-P-A-X-X-[H/Q] (Figure 3). This signature is also conserved in other green algal proteins (Cre_24 and Sco_01) and in the protein Ppa_46 from *P. patens*. Therefore, the "green" LI818 proteins cannot recognize chlorophyll b and lutein in the same way that the spinach CAB binds these molecules [42].

Altogether, these analyses support the conservation of the chlorophyll a binding sites in LI818 proteins. However, these proteins can probably not recognize chlorophyll b and lutein molecules as observed in the spinach CAB structure [42], due to the large displacement of helix α2 (Figure 4). These structural features raise questions about the nature of the pigments bound in the conserved helix α2 region of the LI818 proteins. In heterokont CBPs, these changes in topology could be related to the potential binding of chlorophyll c and fucoxanthin molecules, easily explaining the differences with the spinach CAB; in contrast, this hypothesis is not relevant for "green" LI818 CBPs, which are expected to bind chlorophyll b and lutein molecules. In addition, this alteration of helix α2 is similar in "brown" and "green" LI818 proteins, supporting that these proteins have diverged from a close, common ancestor. This suggests that green LI818 proteins may have evolved in an ancestral chlorophyll c/fucoxanthin-containing organism and were possibly acquired by green algae later.

### The origin of LI818 proteins in different lineages

**Figure 5:**
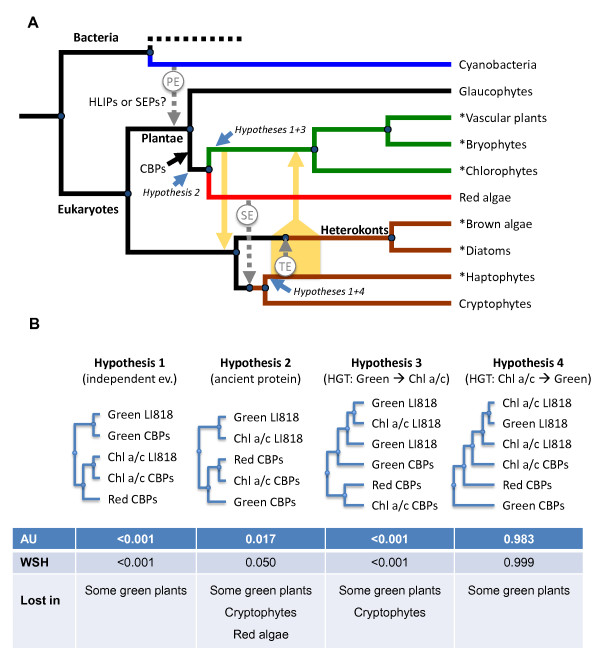
**Schematic representation of the possible evolution of CBPs from Chl a/b-, Chl a-, and Chl a/c-containing organisms**. A: Simplified representation of the nuclear phylogeny of photosynthetic organisms according to Sanchez-Puerta and Delwiche [70,71]. Hypotheses 1, 2, 3, and 4 as well as blue arrows indicate possible origins of LI818 proteins (see B and text). Orange arrows designate possible horizontal gene transfers according to hypotheses 3 and 4. Names marked with "*" indicate branches with known LI818 proteins. PE = primary endosymbiosis, SE = secondary endosymbiosis, TE = tertiary endosymbiosis. B: Different hypotheses regarding the position of LI818 proteins and associated p-values using the Approximately Unbiased test (AU) and the Weighted Shimodaira-Hasegawa test (WSH). "Lost in" indicates lineages in which, based on currently available sequence data, we would assume LI818 proteins to have been lost according to these hypotheses. Hypothesis 1 assumes that LI818 originated independently in green plants and chlorophyll a/c-containing organisms; Hypothesis 2 assumes that LI818 proteins evolved before the separation of the red- and green lineage; Hypothesis 3 assumes a transfer of genes from the green lineage to a common ancestor of heterokonts and haptophytes (Chl a/c) during the cryptic endosymbiosis event proposed by Moustafa et al. [28]; Hypothesis 4 assumes a horizontal gene transfer from an ancestral chlorophyll a/c-containing organism to an early member of the green lineage.

This latter observation prompted us to reexamine the origin of LI818 proteins in greater detail. The absence of CBP proteins in cyanobacteria and in an extensive EST library of the glaucophyte *Cyanophora paradoxa *[43] indicates that ancestral CBPs evolved after the separation of glaucophytes from red algae and green plants (Figure 5A). The presence of red-, haptophyte-, and heterokont CBPs within the same group is in agreement with the hypothesis that these proteins originate from a photosynthetic organism from the red lineage, and were transferred during secondary endosymbiosis. This event, however, cannot explain the presence of green LI818 proteins in a branch of the tree (Figure 1) otherwise comprising purely red- and chlorophyll a/c-containing algae. Interestingly, very similar observations were previously made for another group of proteins: Frommelt et al. [27] found that 5 of 16 protein sequences from heterokonts, haptophytes, and cryptophytes involved in carotenoid synthesis were closest to prasinophytes and not to red algal sequences. Although genes involved in carotenoid biosynthesis and chlorophyll-binding proteins are not related, they are both involved in photosynthesis. Notably, the enzymes violaxanthin de-epoxidase and zeaxanthin epoxidase, both of which were closest to the green linage in the study of Frommelt et al. [27], are part of the xanthophyll cycle, and thus an important actor in the regulation of NPQ, just as it has been suggested for LI818 proteins [18,23,25].

To go deeper in the analysis, we tested four different hypotheses regarding the phylogenetic position of the LI818 proteins by manually rearranging trees and performing statistical analyses. The first hypothesis assumes independent but convergent evolution of LI818 proteins in the "green" and the "brown" lineages. Our results show this scenario to be highly unlikely (Hypothesis 1, p-values < 0.00001; Figure 5B). While our model cannot account for environmental factors that might favor such a scenario, the aforementioned structural data provide additional strong arguments against this hypothesis. The second possibility was that LI818 proteins may be ancestral CBPs that have evolved in a common ancestor of the red and green lineage [20] and have subsequently been lost in many embryophytes and probably also in red algae (Figure 5B, Hypothesis 2). In this case the position of the green LI818 proteins in our phylogenetic tree should be in reality at the root of the green branch. The fact that LI818 proteins were not found in red algae or cryptophytes could be explained either by insufficient sequence data for these organisms or by a secondary loss of these proteins. Based on the statistical analysis of a tree manually rearranged to fit this hypothesis, this option cannot be clearly ruled out (p = 0.017 and 0.05 with the AU and WSH tests, respectively).

A third hypothesis was previously proposed by Peers et al. [18] and consists in a horizontal transfer of green LI818 genes to a common ancestor of heterokonts and haptophytes during a cryptic primary endosymbiosis event. Such an event is believed to have taken place before secondary endosymbiosis [28], and could explain the absence of LI818 proteins in red algae but not in cryptophytes. Under the assumption that LI818 proteins were transferred during this event, we would expect LI818 proteins to branch early from the green lineage. A tree manually rearranged to fit this hypothesis received p-values < 0.001 in both AU and WSH tests (Figure 5B, Hypothesis 3). Hence, based on our analysis, this option seems highly unlikely. In addition, several studies have recently provided independent evidence against the "Chromalveolate hypothesis" [44], favoring distinct rhodobiont endosymbioses in chlorophyll a/c-containing algae [30,45,46]. Under this evolutionary scenario, it seems unlikely that a cryptic green algal endosymbiosis could have preceded the plastidic secondary endosymbiosis in each lineage of chlorophyll a/c-containing algae.

The last hypothesis, which was prompted by our examination of the structure of LI818 proteins, was that the first members of this family could have evolved in a chlorophyll a/c-containing organism after secondary endosymbiosis, and that a horizontal gene transfer could have taken place to an early green organism (Figure 5B, Hypothesis 4). Based on the close proximity of green and haptophyte LI818 proteins in Figure 1, such a transfer could have involved an ancestral haptophyte. Molecular clock studies date the split of the red- and green lineage to the late mesoproterozoic, over 900 million years ago (Mya) [47,48], and secondary plastid endosymbiosis is assumed to have occurred shortly thereafter [47]. The same studies date diversification of the green lineage to approximately 730 to 700 Mya, respectively, leaving a time-frame of about 200 million years during the early neoproterozoic for such an event to have taken place. This hypothesis does not assume any losses of LI818 proteins except in parts of the green lineage, and best fits our phylogenetic tree because all LI818 proteins constitute a subfamily within the group of chlorophyll a/c-containing and red algal proteins (p-values of 0.983 to 0.999 with AU and WSH tests respectively). In accordance with this, the phylogenetic trees obtained for enzymes involved in carotenoid biosynthesis [27] could also be interpreted in the same sense, since, just as for CBPs, several proteins from green algae (*e.g. *phytoene desaturase, isopentenyl diphosphate:dimethylallyl diphosphate isomerase, zeaxanthin epoxidase, violaxanthin de-epoxidase) branched within a group of haptophyte and/or heterokont sequences, and not as a sister group. Gene acquisition by green algae is not unprecedented. The genomic analysis of *Micromonas *unraveled the transfer of bacterial genes involved in biosynthesis of peptidoglycan [49], and this alga has also acquired several brown algal genes responsible for mannitol synthesis [31].

Although the evidence presented in this study is not strong enough to definitely reject Hypothesis 2 (Figure 5B), our findings provide a strong indication against the putative transfer of LI818 proteins during a cryptic green algal endosymbiosis event preceding red algal secondary endosymbiosis (Hypothesis 3), and favor a scenario with a transfer in the other direction (Hypothesis 4).

## Conclusion

This study shows that *E. siliculosus *contains a wide variety of CBPs from different divisions and with potentially different functions. In all of the examined species, most of the CBPs known to be stress-induced belong to the LI818 family, which is particularly well represented among haptophytes and heterokonts, but also present in several organisms of the green lineage. According to our analyses, the possibility that LI818 proteins may be ancestral CBPs, which have evolved before the separation of the red and the green lineage, remains viable. However, structural alignments and three-dimensional modeling illustrated several elements common to LI818 proteins, which are likely to interfere with their capacity to bind certain chlorophyll b molecules, but may enable the binding of chlorophyll c and/or fucoxanthin. This finding, together with the statistical analysis of our phylogenetic tree, point to a new possibility not dealt with in previous studies, *i.e. *that LI818 proteins may have originated in an ancient chlorophyll a/c-containing organism and could have been later transferred to the green linage. Understanding the evolutionary history of CBPs may also increase our understanding of the evolution of the different eukaryotic lineages as a whole. If our hypothesis is correct, LI818 proteins will not be found in red algae. We thus anticipate that ongoing genome projects such as those of the red algae *Chondrus *and *Porphyra *will confirm this prediction.

## Methods

### Sequence retrieval

Four approaches were taken to select sequences for this study. First, CBPs were searched for in the sequenced genomes of photosynthetic eukaryotes representing the major photosynthetic lineages (Table 1). These searches were carried out using "blastp" and reference sequences representing the four major clades in our phylogeny. All hits (e-value < 10) were submitted to InterProScan [50] using default parameters, and considered CBPs if a "Chlorophyll A-B binding protein" domain corresponding to the PFAM00504/IPR022796 motif was detected. All sequence identifiers, database references, and InterProScan domains are given in Additional file 1. Many of these sequences were included in our phylogenetic analysis; however, in cases where the results obtained for related species were highly similar, only a representative was selected.

In a second step we attempted to fill gaps in our phylogenetic tree, where no or only limited genome sequences were available, by using data from EST libraries. For these searches "tblastn" was used instead of "blastp", and in case of duplicate sequences, the longest EST was chosen. The identified CBP sequences were translated using NCBI OrfFinder (http://www.ncbi.nlm.nih.gov/projects/gorf/). In the case of red algae, only the reduced genome of *C. merolae *was available for public use, and we added EST sequences from *C. crispus *(4,114 ESTs, 3 coding for CBPs; [51]), *P. yezoensis *(20,069 ESTs, 4 coding for CBPs; [52]), and *G. changii *(8,147 ESTs, 7 coding for CBPs; Teo et al., unpublished), all of which were obtained from the NCBI EST database (dbEST). Furthermore, as no genome sequences are available for cryptophytes, the same strategy was applied for an EST library of *Guillardia theta *(15,173 ESTs, 21 coding for CBPs; [53]), and we also took advantage of the availability of five CBP sequences obtained in a previous study by Broughton et al. on *Rhodomonas sp. *CS24 [35].

As we were particularly interested in stress-induced CBPs and the possible role of these CBPs in NPQ, we considered additional sequences relevant for these topics in a third step: seventeen ESTs coding for CBPs from the Antarctic diatom *Chaetoceros neogracile *(total number of ESTs in the library 1,744; [10]), and six previously identified CBPs from *Fucus serratus *and *F. vesiculosus *were selected because transcriptomic data were available for these sequences [10,11,14]; also, three sequences from the diatom *Cyclotella meneghiniana *were included, because these proteins had been shown to be related to NPQ [25].

Finally, several individual sequences were added to our phylogenetic tree in order to facilitate comparisons with previous phylogenetic studies [19,20,36,54], and to include a wider selection of LI818 proteins from the green lineage. For the latter purpose, blast searches were performed in the NCBI nr and EST databases, and the retrieved sequences were treated as described above. We added two sequences from the chlorarachniophyte (Rhizaria) *Bigelowiella natans*, which is thought to have acquired its plastids through an independent secondary endosymbiosis event with a green alga [55]; one sequence from the dinophyte *Karlodinium micrum*; two sequences from the unicellular green algae *Mesostigma viride *(Charophyta) and *Scenedesmus obliquus *(Chlorophyta); one sequence from the haptophyte *Pavlova lutheri*; two sequences from a second strain of *Micromonas sp*. (CCMP490); three sequences from *Ostreococcus sp*. RCC809; one sequence each from *O. tauri *and *V. carteri *(these had also been identified in our analysis of the corresponding genomes); and three sequences from the gymnosperm *Picea sitchensis*. The sequence corresponding to the first available CAB structure from *Pisum sativum *[56] was included to enable homology-based three-dimensional modeling, but was later replaced by the spinach protein mentioned below. A complete list of sequences and accession numbers is available in Additional file 2, and further LI818 proteins are reported in the first paragraph of the results and discussion section, even though they were not included in the phylogenetic analyses due to the high degree of sequence similarity with other represented sequences.

### Phylogenetic analyses

A phylogenetic tree was constructed for all sequences except those of poplar and maize, which were highly similar to those of *A. thaliana*, and those of *O. lucimarinus*, as they were highly similar to *O. tauri*. To this aim, sequences were aligned using MAFFT [57] and the "E-INS-i" strategy with default settings. Automatic alignments were then manually refined using Bioedit 7.0.9.0 [58], taking into consideration only the conserved regions of the proteins for further analyses. A total of 131 residues were manually selected (see Additional file 3). In some cases, this resulted in two sequences with 100% sequence identity, and one of the identical sequences was excluded for further analyses. In parallel, an automatic selection of conserved residues was carried out using the Gblocks algorithm [59]. Even with the least stringent settings, this resulted in the selection of only 53 residues. Phylogenetic analyses based on these 53 residues yielded similar tree topologies as the manual selection (data not shown). CBPs and FCPs were considered stress-induced if they were identified as such in the original publications, or in the case of *E. siliculosus*, if the mean ratio stress/control for hyposaline-, hypersaline-, and oxidative stress reported in our previous study was greater than 2, and the p-value (ANOVA) associated to this change < 0.05 [60].

Maximum likelihood trees were generated using PhyML (bootstrapping with 250 iterations as well as Approximate Likelihood-Ratio testing [61]). We chose the "Blosum62" substitution model allowing gamma-distributed rate heterogeneity over sites (4 categories) as well as invariable sites, as this was shown to best represent our data using ProtTest [62] and the AIC criterion. The results were complemented by bayesian inference analysis using MrBayes [63]. Except for the use of "Blosum62" as prior for the substitution model and gamma-distributed rate heterogeneity in the likelihood model (see above), default parameters were applied. The analysis was run for 8,000,000 generations (samples were taken every 100 generations). At this point, the average standard deviation of split frequencies was stable at approximately 0.03 and decreased no further. The first 25% of samples were discarded as burn in. The PhyML tree was used as a basis to manually introduce targeted modifications in the tree and to run the tests evaluating different evolutionary scenarios on the origin of LI818 proteins. Different trees were tested using treepuzzle [64] to calculate site-wise log-likelihood (same substitution model as above), and CONSEL [65] to perform the Approximately Unbiased (AU, [66]) and the Weighted Shimodaira-Hasegawa tests (WSH).

### Sequence analysis and three-dimensional modeling

A multiple sequence alignment of the LI818-like sequences was generated as described above, and manually refined using BioEdit, on the basis of the crystal structure of the CAB from spinach (PDB code 1RWT; [42]). The chain A of this structure was also used to generate a three dimensional (3D) model of the proteins Cre_23 from *C. reinhardtii *and Esi_02 from *E. siliculosus *using Modeller [67] with default parameters. The multiple sequence alignment and the 3D models were displayed using ESPript [68] and PyMOL [69], respectively.

## List of abbreviations

CAB: chlorophyll a/b-binding protein; CBP: hlorophyll-binding protein; ELIP: early light-induced protein; EST: expressed sequence tag; FCP: fucoxanthin chlorophyll a/c-binding protein; HLIP: high light-induced protein; LHC: light-harvesting complex; NPQ: non-photochemical quenching; SEP: stress-enhanced proteins;

## Authors' contributions

SD, GM and TT conceived the study, together with JC and CB, SD, GM, and TT performed and interpreted the analyses. SD wrote the manuscript together with GM, CB, JC, and TT. All authors approved the final manuscript.

## Supplementary Material

Additional file 1 **Databases and sequence identifiers of CBPs found in the genomes of the organisms listed in Table **1. Each organism is represented by a separate sheet. "SignalP" indicates the presence of a signal peptide. Click here for file

Additional file 2 **Accession numbers of all sequences considered for the phylogenetic tree in Figure **1** (including those removed due to high sequence identity)**. For stress-induced proteins, the PubMed Id (PMID) of the corresponding publication is given. Click here for file

Additional file 3 **Alignment used for the construction of the phylogenetic tree in Figure **1. Click here for file
